# Association of Prenatal Exposure to Persistent Organic Pollutants with Obesity and Cardiometabolic Traits in Early Childhood: The Rhea Mother–Child Cohort (Crete, Greece)

**DOI:** 10.1289/ehp.1409062

**Published:** 2015-04-24

**Authors:** Marina Vafeiadi, Vaggelis Georgiou, Georgia Chalkiadaki, Panu Rantakokko, Hannu Kiviranta, Marianna Karachaliou, Eleni Fthenou, Maria Venihaki, Katerina Sarri, Maria Vassilaki, Soterios A. Kyrtopoulos, Emily Oken, Manolis Kogevinas, Leda Chatzi

**Affiliations:** 1 Department of Social Medicine, Faculty of Medicine, University of Crete, Heraklion, Greece; 2 Department of Environmental Health, National Institute for Health and Welfare, Kuopio, Finland; 3 Laboratory of Clinical Chemistry-Biochemistry, Department of Laboratory Medicine, School of Medicine, University of Crete, Heraklion, Crete, Greece; 4 Institute of Biology, Medicinal Chemistry and Biotechnology, National Hellenic Research Foundation, Athens, Greece; 5 Obesity Prevention Program, Department of Population Medicine, Harvard Medical School and Harvard Pilgrim Health Care Institute, Boston, Massachusetts, USA; 6 Centre for Research in Environmental Epidemiology (CREAL), Barcelona, Spain; 7 Hospital del Mar Research Institute (IMIM), Barcelona, Spain; 8 CIBER Epidemiología y Salud Pública (CIBERESP), Barcelona, Spain; 9 National School of Public Health, Athens, Greece

## Abstract

**Background:**

Prenatal exposure to endocrine-disrupting chemicals such as persistent organic pollutants (POPs) may increase risk of obesity later in life.

**Objective:**

We examined the relation of *in utero* POPs exposure to offspring obesity and cardiometabolic risk factors at 4 years of age in the Rhea mother–child cohort in Crete, Greece (*n* = 689).

**Methods:**

We determined concentrations of polychlorinated biphenyls (PCBs), dichlorodiphenyldichloroethylene (DDE), and hexachlorobenzene (HCB) in first-trimester maternal serum. We measured child weight, height, waist circumference, skinfold thicknesses, blood pressure (BP), blood levels of lipids, C-reactive protein, and adipokines at 4 years of age. Childhood obesity was defined using age- and sex-specific cut points for body mass index (BMI) as recommended by the International Obesity Task Force.

**Results:**

On multivariable regression analyses, a 10-fold increase in HCB was associated with a higher BMI *z*-score (adjusted β = 0.49; 95% CI: 0.12, 0.86), obesity [relative risk (RR) = 8.14; 95% CI: 1.85, 35.81], abdominal obesity (RR = 3.49; 95% CI: 1.08, 11.28), greater sum of skinfold thickness (β = 7.71 mm; 95% CI: 2.04, 13.39), and higher systolic BP (β = 4.34 mmHg; 95% CI: 0.63, 8.05) at 4 years of age. Prenatal DDE exposure was associated with higher BMI *z*-score (β = 0.27; 95% CI: 0.04, 0.5), abdominal obesity (RR = 3.76; 95% CI: 1.70, 8.30), and higher diastolic BP (β = 1.79 mmHg; 95% CI: 0.13, 3.46). PCBs were not significantly associated with offspring obesity or cardiometabolic risk factors.

**Conclusions:**

Prenatal exposure to DDE and HCB was associated with excess adiposity and higher blood pressure levels in early childhood.

**Citation:**

Vafeiadi M, Georgiou V, Chalkiadaki G, Rantakokko P, Kiviranta H, Karachaliou M, Fthenou E, Venihaki M, Sarri K, Vassilaki M, Kyrtopoulos SA, Oken E, Kogevinas M, Chatzi L. 2015. Association of prenatal exposure to persistent organic pollutants with obesity and cardiometabolic traits in early childhood: the Rhea mother–child cohort (Crete, Greece). Environ Health Perspect 123:1015–1021; http://dx.doi.org/10.1289/ehp.1409062

## Introduction

Persistent organic pollutants (POPs) are ubiquitous and persist in the environment, accumulate in high concentrations in fatty tissues, and are biomagnified through the food chain. POPs include synthetic chemicals that were widely used as pesticides [e.g., dichlorodiphenyltrichloroethane (DDT), dichlorodiphenyldichloroethylene (DDE), hexachlorobenzene (HCB)] and in industrial processes [polychlorinated biphenyls (PCBs)] throughout most of the 20th century. Human exposure to POPs occurs primarily through diet, and maternal concentrations of POPs are transmitted to the developing fetus prenatally through the placenta and postnatally via breast milk (Needham et al. 2011). The use of these chemicals is presently banned (PCBs, HCB) or restricted (DDT) (Stockholm Convention on Persistent Organic Pollutants 2009). However, because of their persistence in the environment, the general population is still exposed to these substances at low doses (Porta et al. 2008) and adverse health outcomes related to background levels of exposure are still a concern [World Health Organization (WHO) 2010]. Moreover, several of these compounds act as endocrine-disrupting chemicals (EDCs), which can alter the normal function of endocrine systems in humans and wildlife (Diamanti-Kandarakis et al. 2009).

*In utero* exposure to EDCs has been hypothesized to increase risk of obesity in childhood and into adulthood (Baillie-Hamilton 2002; Diamanti-Kandarakis et al. 2009; La Merrill and Birnbaum 2011). According to the “environmental obesogen hypothesis,” early-life exposure to chemicals that could mimic or block the natural action of endogenous hormones may perturb the mechanisms involved in adipogenesis or energy storage, and thus may increase an individual’s susceptibility to obesity (Grün and Blumberg 2006).

Several longitudinal birth cohort studies have examined the relationship of prenatal exposure to EDCs with child growth and obesity (Cupul-Uicab et al. 2013; Lamb et al. 2006; Mendez et al. 2011; Smink et al. 2008; Tang-Péronard et al. 2014; Valvi et al. 2012, 2014; Verhulst et al. 2009; Warner et al. 2013, 2014). Prenatal DDT and DDE exposure has been positively associated with body mass index (BMI) in infancy (Mendez et al. 2011; Valvi et al. 2014; Verhulst et al. 2009) and later childhood (Tang-Péronard et al. 2014; Valvi et al. 2012; Warner et al. 2014). Findings for the association between prenatal PCB exposure and childhood obesity have been less consistent (Cupul-Uicab et al. 2013; Lamb et al. 2006; Mendez et al. 2011; Tang-Péronard et al. 2014; Valvi et al. 2012, 2014; Verhulst et al. 2009). Prenatal HCB exposure has been positively associated with rapid growth in the first 6 months of life (Valvi et al. 2014) and obesity in infancy (Valvi et al. 2014) and childhood (Smink et al. 2008). Direct comparison of results from previous studies is limited by variations in exposure and outcome assessment and also because associations seem to be modified by other factors such as sex, smoking, or maternal BMI (Mendez et al. 2011; Tang-Péronard et al. 2014; Valvi et al. 2012, 2014; Verhulst et al. 2009; Warner et al. 2014).

Cardiovascular risk factors measured in early childhood such as blood pressure and lipid levels are predictive of adulthood risk for coronary artery disease, suggesting that exposure to these factors early in life may induce changes in arteries that contribute to the development of atherosclerosis (Li et al. 2003; Raitakari et al. 2003). Exposure to environmental chemicals in adulthood has been associated with other cardiovascular traits such as higher blood pressure (La Merrill et al. 2013; Peters et al. 2014) and high serum lipid levels (Aminov et al. 2013; Patel et al. 2012) in adults. However, to our knowledge, there are no studies so far on the effect of prenatal POP exposure on offspring cardiovascular traits other than adiposity.

In the present study, we examined the association of maternal serum HCB, DDE, and PCBs levels in pregnancy with offspring obesity and a range of cardiovascular risk factors [BMI, fat mass, waist circumference, blood pressure, lipids, adiponectin, leptin, and C-reactive protein (CRP)] at 4 years of age in the Rhea mother–child cohort in Crete, Greece.

## Materials and Methods

*Study population*. The Rhea study prospectively examines a population-based sample of pregnant women and their children at the prefecture of Heraklion, Crete, Greece. Methods are described in detail elsewhere (Chatzi et al. 2009). Briefly, female residents (Greek and immigrants) who became pregnant during a period of 1 year starting in February 2007 were contacted and asked to participate in the study. The first contact was made at the time of the first comprehensive ultrasound examination (mean ± SD, 11.96 ± 1.49 weeks), and several contacts followed (6th month of pregnancy, at birth, 9 months, 1 year, and 4 years after birth). To be eligible for inclusion in the study, women had to have a good understanding of the Greek language and be > 16 years of age. Face-to-face structured questionnaires along with self-administered questionnaires and medical records were used to obtain information on several psychosocial, dietary, and environmental exposures during pregnancy and early childhood. The study was approved by the ethics committee of the University Hospital in Heraklion, Crete, Greece, and all participants provided written informed consent after complete description of the study.

Eligible women (*n* = 1,765) were approached during the enrollment period, 1,610 (91%) agreed to participate, and 1,459 singletons (86%) were followed up until delivery. A total of 1,135 blood samples provided by the study participants were analyzed for POP exposure, and 879 children participated at the 4-years follow-up of the study, during which anthropometry and nonfasting blood samples were obtained from 785 children. From those, complete data for POP exposure, follow-up interview, and anthropometric measurements were available for 689 mother–child pairs and thus were eligible for analysis.

*POP exposure*. Maternal serum samples were collected at the first prenatal visit around the 3rd and 4th month of pregnancy in 10-mL silicon gel separator vacutainers (Becton Dickinson), were centrifuged within 2 hr from blood collection at 2,500 rpm for 10 min, and were then stored in aliquots at –80°C until assayed. The POP analyses were performed in the National Institute for Health and Welfare, Chemical Exposure Unit, Kuopio, Finland, with an Agilent 7000B gas chromatograph triple quadrupole mass spectrometer (GC-MS/MS). Pretreatment of serum samples for GC-MS/MS analysis has been described elsewhere (Koponen et al. 2013). Serum concentrations of six individual PCB congeners (118, 138, 153, 156, 170, and 180), HCB, DDT and DDE, and BDE-47 (polybrominated diphenyl ether) were determined. All the results were reported on whole weight and expressed in picograms per milliliter serum, whereas samples below the limit of quantification (LOQ) were assigned the value 0.5 × LOQ. LOQ was 6 pg/mL for PCB-118 and PCB-156; 10 pg/mL for HCB, DDE, PCB-138, PCB-153, PCB-170, PCB-180, and BDE-47; and 50 pg/mL for DDT. We chose to use wet-weight levels for the POPs but adjusted for fasting maternal serum triglycerides (mean ± SD, 128.4 ± 56.6 mg/dL) and cholesterol (208.9 ± 44.5 mg/dL) as continuous variables in all multivariable models to minimize potential biases associated with automatic lipid adjustment (Schisterman et al. 2005). The percentage of samples with levels of DDT above the LOQ was 35.3%. For PCB-156 and BDE-47, 53.9% and 23.1% were above the LOQ, respectively. Because of high percentages of samples below the LOQ, DDT and BDE-47 were not used in the statistical analyses. PCB-156 was included in analyses of summed PCB concentrations, but analyses were repeated after excluding this pollutant with no change in the results (data not shown). POPs were treated as continuous variables on a log_10_ scale. We calculated total PCB concentrations by summing the concentrations of the six individual PCB congeners. We estimated associations using separate models for DDE, HCB, and the sum of PCBs and using multi-pollutant models that included all three exposures.

*Child anthropometry*. Age- and sex-specific *z*-scores for weight at birth and at 6 months of age were calculated using internally generated growth reference curves. Rapid growth from birth to 6 months of age was defined as a *z*-score weight gain > 0.67 SD (Monteiro and Victora 2005). Children with a *z*-score weight gain ≤ 0.67 SD were characterized as slow/average growers and constituted the reference group.

At 4 years of age, body weight was measured once by a digital scale (SecaBellisima 841) to the nearest 0.1 kg with subjects standing without shoes and in light clothing. Height was measured to the nearest 0.1 cm with the use of a commercial stadiometer (Seca 213). The measurement of height was conducted without shoes and in a standing position with children keeping their shoulders in a relaxed position, their arms hanging freely and with their head aligned in Frankfort horizontal plane. Child BMI (weight/height^2^)was used to estimate age- and sex-specific *z*-scores using the mean of the population under study. We categorized weight status at 4 years according to the International Obesity Task Force (IOTF) definitions (Cole and Lobstein 2012).

Waist circumference (WC) was measured in duplicate to the nearest 0.1 cm in the standing position, at the high point of the iliac crest at the end of a gentle expiration, using a flexible tape measure (Seca 201). Because there are no international WC percentile cut-off points defined to identify central adiposity in children or adolescents, we used a Greek national reference to define central adiposity as WC ≥ 90th percentile for age and sex (Linardakis et al. 2010).

Triceps, subscapular, suprailiac, and quadriceps skinfold thicknesses were measured according to standardized techniques to the nearest 0.1 mm using a calibrated Harpenden caliper (Harpenden HSK-BI, CE-0120). Three complete sets of measurement were taken consecutively, and the mean was used as the representative value for each site. Fat mass was expressed as the sum of the four aforementioned skinfolds.

*Child cardiometabolic risk factors*. After 5 min rest in the seated position, trained research assistants measured systolic (SBP) and diastolic (DPB) blood pressure levels on the child’s right arm using an automatic oscillometric device (Pro Care 400, Dinamap) with a cuff of appropriate size for arm circumference. Five measurements were made at 1-min intervals, and the average of all measurements was used for analysis (Gillman and Cook 1995). Nonfasting blood samples were collected from the children at the end of the visit in 10-mL vacutainer tubes with the use of standard procedures, with samples immediately spun, separated, and frozen at –80°C. Analysis of lipids [total cholesterol and high-density lipoprotein cholesterol (HDL-C)] was performed by standard enzymatic methods (Medicon) on an automatic analyzer (AU5400 high-volume chemistry analyzer; Olympus America Inc.). Leptin and adiponectin were measured by enzyme-linked immunosorbent assays (leptin: DLP00, R&D Systems Inc.; and adiponectin: KHP0041, Invitrogen Corporation) on an automatic analyzer (MAGO plus, Diamedix). CRP levels were measured with a high-sensitivity homogenous immunoassay (ORS 6199; Beckman Coulter) on an automatic analyzer (AU5400 high-volume chemistry analyzer; Olympus America Inc.), and abnormal levels were defined as > 3 mg/L (Ford et al. 2005). All inter- and intra-assay coefficients of variation were < 5.5%.

*Statistical analysis*. Descriptive analyses of the study population characteristics, exposures, and outcomes were conducted. Generalized additive models (GAMs) were applied to explore the shape of the relationships between POPs in maternal serum and outcomes under study. These models indicated clear linear relationships (*p*-gain > 0.05). Multivariate generalized linear models for binary outcomes [with log link, Poisson distribution, and robust variance estimator (Zou 2004)] were used to estimate relative risks (RRs) and 95% confidence intervals (CIs) for the associations between the POP exposures and the outcome variables. Multivariate linear regression models were used to examine the association between POPs in maternal serum with continuous outcomes.

We used directed acyclic graphs (DAGs) to choose potential confounders for model adjustment. The set of variables selected for adjustment were maternal prepregnancy BMI (kilograms per meter squared), maternal age at birth (years), parity (primiparous, multiparous), maternal educational level [low (≤ 9 years of mandatory schooling), medium (> 9 years of schooling up to attending postsecondary school education), high (attending university or having a university/technical college degree)], smoking during pregnancy (never, ever), breastfeeding duration (months), and birth weight. This list of variables plus study child’s sex, gestational age, and exact age at outcome assessment, maternal serum levels of triglycerides and total cholesterol were the covariates selected *a priori* for model adjustment. Additionally, we assessed whether maternal weight before pregnancy (kilograms), gestational weight gain [GWG (kilograms)], ethnic origin (Greek, non-Greek), residence (urban, rural), alcohol consumption during pregnancy (yes, no), delivery type (vaginal delivery, cesarean section), delivery hospital (private, public), marital status (married, not married), and working during pregnancy (yes, no) had further influence on the effect estimates. If inclusion of a variable altered the contaminant coefficient by ≥ 10%, we retained the variable in the final set of covariates. The same set of covariates was used for all exposure–outcome combinations. None of the additionally tested variables were selected with this strategy. We looked for heterogeneity in associations related to child sex (male, female), maternal smoking (never, ever), and GWG categories [inadequate, adequate, excessive according to the Institute of Medicine (IOM) guidelines of 2009 based on prepregnancy BMI (Rasmussen and Yaktine 2009)], or maternal prepregnancy BMI status (< 25 kg/m^2^, ≥ 25 kg/m^2^) by including interaction terms in the models for child *z*-BMI and by stratifying the sample. Statistical significance was defined by an alpha level of 0.10 for interaction terms and of 0.05 for all other effect estimates. We performed sensitivity analysis to explore remaining confounding. In particular, we repeated the analyses excluding children who had been born preterm (< 37 gestational weeks) or at low birth weight (< 2,500 g). Because child BMI could be an intermediate in the effect of prenatal POP exposure on waist circumference, leptin, blood pressure, and lipids, we performed sensitivity analyses with adjustment for child BMI. Further, we repeated all analyses without adjustment for birth weight. Analyses were conducted using STATA software, version 13.0 (StataCorp).

## Results

Participating mothers were predominantly Greek, had a mean (± SD) age of 29.9 ± 5.0 years, about half of them had medium educational level (51%) and were multiparous (57%) (Table 1). Before pregnancy, 20% of mothers were overweight, almost all mothers (87%) initiated breastfeeding, and the mean duration of breastfeeding was 4.1 ± 4.3 months. Table S1 in the Supplemental Material shows that mothers without offspring follow-up data were more likely to be younger, less educated, and not Greek.

**Table 1:** Maternal and child characteristics, Rhea mother–child cohort, Crete, Greece (*n* = 689).

Characteristic	*n*	Percent or mean ± SD
Maternal characteristics
Maternal age (years)	681	29.9 ± 5.0
< 20	18	2.6
≥ 20–30	276	40.5
≥ 30–40	368	54.1
≥ 40	19	2.8
Prepregnancy BMI (kg/m^2^)	675	24.4 ± 4.7
Underweight (< 18.5)	22	3.3
Normal (≥ 18.5–25)	437	64.7
Overweight (≥ 25–30)	137	20.3
Obese (≥ 30)	79	11.7
Weight gain during pregnancy (kg)	566	13.8 ± 5.6
Ethnic origin
Other	37	5.4
Greek	648	94.6
Education
Low	105	15.5
Medium	347	51.2
High	226	33.3
Parity
Primiparous	284	42.9
Multiparous	378	57.1
Smoking during pregnancy
Never	432	64.7
Ever	236	35.3
Type of delivery
Vaginal	339	49.4
Cesarean	347	50.6
Breastfeeding (months)	663	4.1 ± 4.3
Never	85	12.8
Ever	578	87.2
BMI at child’s 4 years	662	25.4 ± 4.9
Underweight (< 18.5)	20	3.0
Normal (≥ 18.5–25)	337	50.9
Overweight (≥ 25–30)	201	30.4
Obese (≥ 30)	104	15.7
Child characteristics at birth
Sex
Boy	358	52.0
Girl	331	48.0
Birth weight (g)	686	3,205 ± 451.7
Gestational age (completed weeks)	686	38.2 ± 1.5
Preterm (< 37 weeks)
Yes	84	12.2
No	602	87.8
Low birth weight
*Yes*	30	4.4
*No*	656	95.6
Rapid growth
Yes	203	35.2
No	374	64.8
Child characteristics at 4 years
Weight at 4 years (kg)	689	18.2 ± 3.0
Height (cm)	689	105.2
BMI (kg/m^2^)	689	16.4 ± 1.9
Underweight	15	2.2
Normal	527	76.5
Overweight	99	14.4
Obese	48	6.9
Triceps skinfold thickness (mm)	657	10.33 ± 2.77
Subscapular skinfold thickness (mm)	661	6.93 ± 2.61
Suprailiac skinfold thickness (mm)	643	7.90 ± 3.86
Quadriceps skinfold thickness (mm)	621	15.54 ± 5.40
Sum of skinfolds (mm)	668	39.1 ± 14.2
Waist circumference (cm)	683	53.6 ± 4.7
Systolic blood pressure (mmHg)	552	90.3 ± 8.1
Diastolic blood pressure (mmHg)	552	53.6 ± 5.4
Total cholesterol (mg/dL)	588	157.1 ± 28.6
HDL cholesterol (mg/dL)	588	47.1 ± 10.6
LDL cholesterol (mg/dL)	588	96.6 ± 25.5
C-reactive protein (mL/dL)	540	0.2 ± 0.4
Leptin (ng/mL)	580	2.9 ± 3.6
Adiponectin (μL/mL)	581	15.4 ± 8.7

About half of the children (52%) were boys, and 49% were vaginally delivered (Table 1). The prevalence of rapid growth in the first 6 months of life was 35% (*n* = 203). Rapid growers were at higher risk for overweight/obesity at 4 years of age (adjusted RR = 1.94; 95% CI: 1.46, 2.56). At the 4-year follow-up, the mean (± SD) offspring BMI was 16.4 ± 1.9 kg/m^2^. In total, 99 (14%) children were classified as overweight, and an additional 48 (7%) were obese at 4 years of age. Mean waist circumference was 53.6 ± 4.7 cm, and a total of 80 (12%) children had a waist circumference ≥ 90th percentile for age.

**Figure 1:**
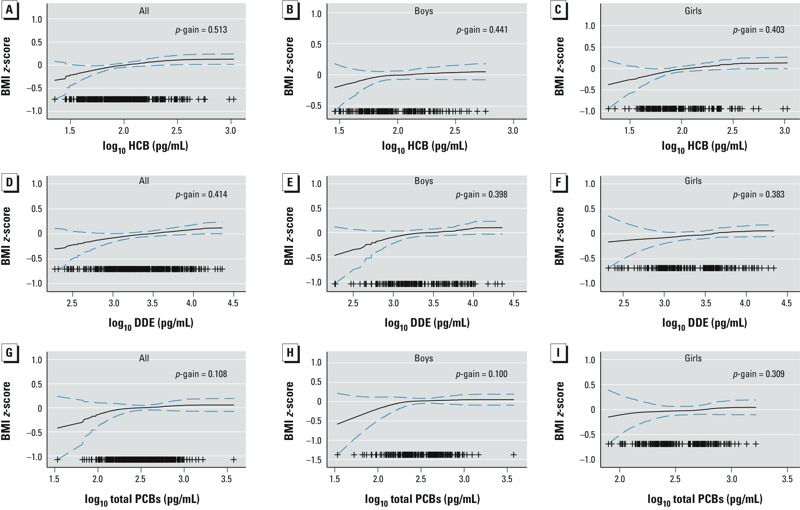
GAMS for adjusted associations (95% CIs) of HCB overall (*A*) and in boys (*B*) and girls (*C*), DDE overall (*D*) and in boys (*E*) and girls (*F*), and PCBs overall (*G*) and in boys (*H*) and girls (*I*) with BMI *z*-score at 4 years. Adjusted for maternal serum triglycerides and cholesterol, maternal age, prepregnancy BMI, parity, maternal educational level, smoking status during pregnancy, breastfeeding duration, child sex, birth weight, gestational age, and exact age at 4-year examination. Plus symbols (+) represent observations.

Maternal POP concentrations are presented in Table 2. The highest concentrations were found for DDE, followed by total PCBs, HCB, and DDT. Spearman correlation coefficients (*p*-value) were 0.48 (< 0.001) for DDE–PCBs, 0.50 (< 0.001) for DDE–HCB, and 0.64 (< 0.001) for PCBs–HCB. Mothers who were older, multiparous, and had higher prepregnancy BMI had higher mean POP levels in early pregnancy (see Supplemental Material, Table S2). Prenatal DDE and HCB levels were similar between women whose offspring had outcome measurements and those who did not, whereas PCB levels were lower in women without offspring follow-up data (see Supplemental Material, Table S1). GAMs examining the shape of the relationships of HCB, DDE, and PCBs with child *z*-BMI at 4 years showed no significant departures from linearity overall and separately in boys and girls (Figure 1).

**Table 2:** First-trimester maternal serum POP levels (pg/mL, *n* = 689), Rhea mother–child cohort, Crete, Greece (*n* = 689).

Contaminants	GM (95% CI)	Minimum	Maximum	Percentile			LOQ	Percent above LOQ
				25th	50th	75th		
HCB	89.4 (85.6, 93.2)	19.5	1330.5	61.7	82.5	116.7	10	100
DDE	2036.2 (1910.2, 2170.5)	181.6	23175.4	1187.6	1981.2	3514.1	10	100
PCB-118	17.1 (16.4, 17.9)	3^*a*^	143.6	12.0	17.7	25.1	6	96.8
PCB-153	124.4 (119.0, 130.2)	13.0	1348.9	85.5	125.2	188.1	10	100
PCB-138	66.5 (63.5, 69.6)	5^*a*^	742.8	45.3	67.8	102.0	10	99.7
PCB-156	6.0 (5.7, 6.4)	3^*a*^	81.5	3^*a*^	6.4	10.7	6	53.9
PCB-180	67.0 (63.7, 70.5)	5^*a*^	979.6	44.1	66.3	104.5	10	99.7
PCB-170	33.4 (31.6, 35.2)	5^*a*^	542.3	22.0	33.7	53.4	10	96.2
Total PCBs	319.1 (304.9, 333.9)	34.0	3758.3	216.7	319.4	483.1
^***a***^Value is LOQ/2.								

Table 3 shows adjusted results for maternal POP levels in relation to offspring obesity-related outcomes. Because models were run with POPs on the log_10_ scale, each unit of increase means a 10-fold increase in the concentration of each contaminant. Prenatal HCB concentrations were positively but not significantly associated with rapid growth in the first 6 months (adjusted RR = 1.94; 95% CI: 0.99, 3.77). At 4 years of age, prenatal HCB concentration was associated with about one-half SD higher BMI *z*-score (adjusted β = 0.49; 95% CI: 0.12, 0.86), and a higher prevalence of obesity (adjusted RR = 8.14; 95% CI: 1.85, 35.81); however, given the small number of children classified as obese (*n* = 32, 6%), the estimates were imprecise, as shown by the wide confidence intervals. Increased *in utero* exposure to HCB was also significantly associated with WC ≥ 90th percentile (adjusted RR = 3.49; 95% CI: 1.08, 11.28), and with a higher sum of skinfold thickness (adjusted β = 7.71 mm; 95% CI: 2.04, 13.39) at 4 years of age. DDE was also associated with an increase in the BMI *z*-score (adjusted β = 0.27; 95% CI: 0.04, 0.5) and higher prevalence of abdominal obesity (adjusted RR = 3.76; 95% CI: 1.70, 8.30) at 4 years of age. In general, PCBs were positively but not significantly associated with offspring obesity outcomes (Table 3).

**Table 3:** Associations of *in utero* POP exposures with early rapid growth and obesity-related outcomes at 4 years of age, Rhea mother–child cohort, Crete, Greece.

Outcome	Cases (%)/total	Exposure^*a*^	Adjusted model^*b*^
Rapid growth 0–6 months^*c*^ [RR (95% CI)]	157 (34.3)/458	HCB	1.94 (0.99, 3.77)
		DDE	1.33 (0.89, 1.99)
		PCBs	1.20 (0.59, 2.46)
BMI *z*-score at 4 years [β (95% CI)]	531	HCB	0.49 (0.12, 0.86)
		DDE	0.27 (0.04, 0.51)
		PCBs	0.21 (–0.20, 0.63)
Obesity at 4 years [RR (95% CI)]	32 (6.0)/531	HCB	8.14 (1.85, 35.81)
		DDE	3.80 (1.19, 12.14)
		PCBs	3.91 (0.49, 31.27)
WC ≥ 90th percentile^*d*^ [RR (95% CI)]	56 (10.7)/526	HCB	3.49 (1.08, 11.28)
		DDE	3.76 (1.70, 8.30)
		PCBs	2.08 (0.65, 6.71)
Sum of skinfolds (mm) at 4 years [β (95% CI)]	515	HCB	7.71 (2.04, 13.39)
		DDE	2.75 (–0.86, 6.35)
		PCBs	5.74 (–0.68, 12.16)
^***a***^POP concentrations are log_10_ transformed (pg/mL). ^***b***^Adjusted for maternal serum triglycerides and cholesterol, maternal age, prepregnancy BMI, parity, maternal educational level, smoking status during pregnancy, breastfeeding duration, child sex, birth weight, gestational age, and exact age at 4-year examination. ^***c***^Rapid growth models are adjusted for maternal serum triglycerides and cholesterol, maternal age, prepregnancy BMI, parity, maternal educational level, smoking status during pregnancy, breastfeeding duration, and child sex and gestational age. ^***d***^WC ≥ 58.6 cm.			

Table 4 presents the associations between maternal POP exposure in pregnancy and offspring cardiometabolic risk factors at 4 years of age. Higher SBP was observed for increasing levels of HCB (adjusted β = 4.34 mmHg; 95% CI: 0.63, 8.05), whereas increasing levels of DDE were associated with higher DBP (adjusted β = 1.79 mmHg; 95% CI: 0.13, 3.46). HCB was also associated with DBP, and DDE was also associated with SBP, though the observed associations were not significant. Maternal HCB and DDE serum levels were positively associated with child blood leptin concentrations at 4 years of age (adjusted β = 2.15 ng/mL; 95% CI: 0.42, 3.89; adjusted β = 1.21 ng/mL; 95% CI: 0.16, 2.27, respectively). Associations attenuated and were no longer significant when we adjusted for child BMI at age of outcome assessment (adjusted β = 1.01 ng/mL; 95% CI: –0.21, 2.23; adjusted β = 0.47 ng/mL; 95% CI: –0.28, 1.21, respectively). Maternal HCB, DDE, and PCB levels were positively associated with elevated offspring CRP levels at 4 years of age, although the observed associations did not reach statistical significance (Table 4). However, only a small number of children had abnormal CRP levels (*n* = 46, 11%). Child lipids (total cholesterol and HDL) and adiponectin levels were not associated with any of the measured POPs (see Supplemental Material, Table S3). Estimates from the multi-pollutant model including DDE, HCB, and PCBs indicate stronger associations with HCB when adjusted for the other two exposures, and comparisons with the single-pollutant model estimates suggest that associations with PCBs were subject to the greatest amount of positive confounding by the other exposures (see Supplemental Material, Table S4).

**Table 4:** Associations of *in utero* POPs exposure with cardiometabolic risk factors at 4 years of age, Rhea mother–child cohort, Crete, Greece.

Outcome	Cases (%)/total	Exposure^*a*^	Adjusted model^*b*^
Systolic blood pressure (mmHg) [β (95% CI)]	427	HCB	4.34 (0.63, 8.05)
		DDE	2.31 (–0.07, 4.69)
		PCBs	2.16 (–2.03, 6.34)
Diastolic blood pressure (mmHg) [β (95% CI)]	427	HCB	2.48 (–0.13, 5.09)
		DDE	1.79 (0.13, 3.46)
		PCBs	–0.49 (–3.43, 2.45)
C-reactive protein > 3 mg/L [RR (95% CI)]	46 (11.3)/409	HCB	2.88 (0.86, 9.64)
		DDE	2.23 (0.94, 5.29)
		PCBs	4.50 (0.89, 22.76)
Leptin (ng/mL) [β (95% CI)]	440	HCB	2.15 (0.42, 3.89)
		DDE	1.21 (0.16, 2.27)
		PCBs	1.55 (–0.42, 3.52)
^***a***^POP concentrations are log_10_ transformed (pg/mL). ^***b***^Adjusted for maternal serum triglycerides and cholesterol, maternal age, prepregnancy BMI, parity, maternal educational level, smoking status during pregnancy, breastfeeding duration, and child sex, birth weight, gestational age, and exact age at 4-year examination.			

We found no evidence of effect modification for BMI *z*-score at 4 years (see Supplemental Material, Table S5) or any of the other outcomes (data not shown) by child sex, smoking status during pregnancy, prepregnancy BMI, or weight gain during pregnancy (all *p*-interaction > 0.10). To evaluate the influence of breastfeeding on rapid growth, we performed a stratified analysis by breastfeeding duration, which showed no substantial differences in observed estimates (data not shown). Sensitivity analyses excluding preterm newborns (< 37 gestational weeks; *n* = 66–45) and infants with low birth weight (i.e., < 2,500 g; *n* = 23–18) did not meaningfully change our results (see Supplemental Material, Table S6). Estimates were also not meaningfully changed by the exclusion of birth weight from the models. Inclusion of child BMI at 4 years in the models for WC slightly decreased the effect estimates, but did not influence their statistical significance (data not shown). Finally, adjusting models of associations for blood pressure and lipids by child BMI at 4 years did not change the results substantially (data not shown).

## Discussion

In this population-based birth cohort study, *in utero* exposure to HCB and DDE was associated with childhood obesity and higher offspring blood pressure levels at 4 years. To our knowledge, this is the first study examining the relationship between prenatal exposure to organochlorine chemicals and child cardiometabolic risk factors other than obesity.

We found that higher prenatal HCB and DDE concentrations were significantly associated with increased BMI *z*-scores and adiposity at 4 years of age. Our findings for HCB are consistent with previous studies that reported positive associations with offspring obesity at 14 months (Valvi et al. 2014) and 6 years (Smink et al. 2008). On the contrary, Delvaux et al. (2014) reported no significant association of HCB measured in cord blood and markers of abdominal obesity at 7–9 years. Low-level prenatal DDE exposure has also been linked to elevated BMI at 14 months of age (Mendez et al. 2011; Valvi et al. 2014) and at 3 years of age (Verhulst et al. 2009), 6.5 (Valvi et al. 2012) and 9 years of age (Warner et al. 2014), and to markers of abdominal fat in girls at 7–9 years (Delvaux et al. 2014). However, recent studies in populations with higher exposures reported null associations between prenatal DDE exposure and BMI at 12 months (Garced et al. 2012), 18 months (Cupul-Uicab et al. 2010), and 7 years of age (Cupul-Uicab et al. 2013; Warner et al. 2013), suggesting a nonmonotonic dose–response relationship (Vandenberg et al. 2012). Potential effect modification by sex and other factors found in previous studies (Cupul-Uicab et al. 2013; Mendez et al. 2011; Tang-Péronard et al. 2014; Valvi et al. 2012; Warner et al. 2014) was not supported in our study, perhaps due to smaller population subgroups, and should be further explored in larger populations. We did not find an association of prenatal PCBs exposure with child adiposity, in line with other studies reporting null associations between prenatal PCB exposure and elevated BMI (Cupul-Uicab et al. 2013; Delvaux et al. 2014; Mendez et al. 2011; Valvi et al. 2014). Although Verhulst et al. (2009) reported a very small increase in BMI *z*-scores associated with PCB exposure in children 1–3 years old and Tang-Péronard et. al (2014) an association with BMI for 7-year-old girls, the preponderance of evidence does not suggest a strong relationship between prenatal PCB exposure and later adiposity. Nevertheless, in this study, estimates from multi-pollutant models suggest that associations with PCBs might be positively confounded by other POPs exposures.

The mechanisms by which prenatal exposure to POPs such as DDE and HCB might be related to increased body fatness are still not clear (Tang-Péronard et al. 2011). *In vitro* studies confirm that DDE exposure at environmentally relevant levels increases proliferation (Chapados et al. 2012) and differentiation (Moreno-Aliaga and Matsumura 2002) of preadipocytes. First, it is possible that obesogenic effects of POPs are mediated by sex steroid dysregulation. DDE is both an estrogen receptor agonist and androgen receptor antagonist, whereas HCB has been suggested to be an androgen receptor and an estrogen-related receptor antagonist (Li et al. 2008). Knockout models of sex steroid pathway components confirm that sex steroids are involved in adipocyte hypertrophy and hyperplasia (Heine et al. 2000; Murata et al. 2002). Second, DDT has been associated with increased mRNA expression of the peroxisome proliferator–activated receptor γ (PPARγ) (Moreno-Aliaga and Matsumura 2002), which constitutes a major regulator of adipogenesis; it is expressed primarily in adipose tissue, and its activation promotes adipocyte differentiation as well as the induction of lipogenic enzymes (Diamanti-Kandarakis et al. 2009). Third, it is possible that organochlorine compounds mediate their obesogenic effect through central integration of energy balance (Grün and Blumberg 2009). We found a positive association between prenatal HCB and DDE levels and child leptin levels, a hormone heavily involved in the control of energy balance (Yu et al. 1997). Consistent with our results, *in vitro* studies have shown that exposure of mature adipocytes to DDE resulted in increased leptin release (Howell and Mangum 2011). Further studies are needed to better understand how prenatal and early-life exposure to obesogenic chemicals, such as organochlorines, can program exposed individuals to gain weight and what role modulation of hormone receptor expression or activity early in life plays in this process.

Prenatal HCB and DDE levels were associated with higher child blood pressure at preschool age. Exposure to environmental chemicals, such as PCBs and DDE, has been associated with higher blood pressure (Goncharov et al. 2011; La Merrill et al. 2013; Lind et al. 2014; Peters et al. 2014) in adults. An association between exposure to HCB and increased heart weight has been reported in mice (Arnold et al. 1986), and exposure to other pollutants such as dioxins and PCBs has also been associated with hypertension in animal studies (Kopf et al. 2008; Lind et al. 2004). Despite the evidence showing an association with cardiovascular parameters, animal models elucidating the mechanisms underlying these effects are limited.

The POPs levels in the study population were close to but generally lower than those of other pregnant populations. Exposure to HCB (median, 0.08 μg/L) was much lower than the median exposure in other studies [Valvi et al. (2012) 0.67 μg/L, Cupul-Uicab et al. (2013) 0.24 μg/L, and Smink et al. (2008) 0.68 μg/L]. Similarly DDE (1.98 μg/L) levels were much lower compared with those reported by Cupul-Uicab et al. (2013) in the United States (24.59 μg/L) but somewhat higher than those of another European study (1.06 μg/L) published by Valvi et al. (2012). PCBs were also lower (0.32 μg/L) than previously reported levels in pregnant women [Valvi et al. (2012) 0.75 μg/L; Cupul-Uicab et al. (2013) 2.74 μg/L]. Therefore, it is possible that relations observed within our study population estimate the association of these chemicals with child health outcomes only within low exposure levels.

Strengths of the present study include the population-based prospective design, the relatively large sample size compared to previous studies and the detailed childhood body fat and cardiometabolic measurements. The fairly homogenous population with regard to factors such as diet, breastfeeding, country of origin, and socioeconomic status can reduce uncontrolled confounding. The levels of attrition in the Rhea cohort are similar to those found in other birth cohort studies (Guxens et al. 2012; Jaddoe et al. 2012), though we found no evidence of differences in prenatal HCB and DDE levels between participants and nonparticipants. We were able to measure levels of several POPs in first-trimester maternal serum using state-of-the-art laboratory techniques, and we were able to assess the adverse effect of combined exposure to several contaminants. However, our study has also some limitations. We could not separate the potential effects of postnatal exposures because we have not measured POPs at other time points, but were able to control for these possible effects by taking into account breastfeeding duration. We also cannot rule out the possibility that prenatal and/or postnatal exposure to other unmeasured chemicals correlated with POPs may have confounded the associations under study. Although we studied a broad spectrum of outcomes, we did not do multiple comparisons because some of the outcomes examined are intercorrelated (adiposity measurements); therefore, we did not apply Bonferroni-type corrections. Finally, although we incorporated extensive information on potential social and environmental factors that are associated with childhood adiposity and cardiometabolic risk factors, we acknowledge that residual confounding from unmeasured covariates such as parental income or social class is still possible.

## Conclusions

This study suggests that prenatal exposure to low levels of DDE and HCB may be associated with greater offspring adiposity and higher blood pressure levels in early childhood. These findings have important public health implications because of the high and increasing prevalence of childhood obesity and ubiquity of POPs exposure. Our future research in the Rhea cohort will examine whether these compounds are associated with alterations in growth at late childhood and puberty onset.

## Supplemental Material

(430 KB) PDF
