# Supplementary material for: Association of Prenatal Exposure to Persistent Organic Pollutants with Obesity and Cardiometabolic Traits in Early Childhood: The Rhea Mother–Child Cohort (Crete, Greece)
Source: Environ Health Perspect. 2015 Apr 24;123(10):1015–21. doi: 10.1289/ehp.1409062 (PMC4590761; doi:10.1289/ehp.1409062)
Supplement: (430 KB) PDF [file ehp.1409062.s001.acco.pdf]

Environ Health Perspect
doi: 10.1289/ehp.1409062

Note to Readers: *EHP* strives to ensure that all journal content is accessible to all readers. However, some figures and Supplemental Material published in *EHP* articles may not conform to 508 standards due to the complexity of the information being presented. If you need assistance accessing journal content, please contact ehp508@niehs.nih.gov. Our staff will work with you to assess and meet your accessibility needs within 3 working days.

Supplemental Material

Association of Prenatal Exposure to Persistent Organic Pollutants with Obesity and Cardiometabolic Traits in Early Childhood: The Rhea Mother–Child Cohort (Crete, Greece)

Marina Vafeiadi, Vaggelis Georgiou, Georgia Chalkiadaki, Panu Rantakokko, Hannu Kiviranta, Marianna Karachaliou, Eleni Fthenou, Maria Venihaki, Katerina Sarri, Maria Vassilaki, Soterios A. Kyrtopoulos, Emily Oken, Manolis Kogevinas, and Leda Chatzi

Table of Contents

Table S1. Maternal and child characteristics of participants and non-participants.

Table S2. Maternal and child characteristics by concentrations of first trimester maternal serum POPs levels (pg/ml), Mother-child cohort “Rhea” in Crete, Greece (n=689).

Table S3. Associations of in utero POPs exposure with child lipids and adiponectin levels at 4 years of age, Mother-child cohort “Rhea” in Crete, Greece.

Table S4. Association between POPs and birth outcomes in multipollutant models including DDE, HCB and PCBs.

Table S5. Associations of in utero POPs exposure with child BMI at 4 years of age in the subgroups defined by infant sex, maternal smoking and maternal prepregnancy BMI status.

Table S6. Associations of in utero POP exposures with the outcomes under study in the total population and without out preterm births and low birth weight neonates.

Table S1. Maternal and child characteristics of participants and non-participants.

Maternal characteristics	Participants n=689	Non-Participants n=619	P- value[a]
Maternal age (years), mean (SD)	29.9±5.0	28.9±5.0	<0.001
Maternal education, n (%)			<0.001
Low	105 (15.5)	166 (27.5)	
Medium	347 (51.2)	301 (49.9)	
High	226 (33.3)	136 (22.5)	
Mother's origin, n (%)			<0.001
Greek	648 (94.6)	533 (86.5)	
Non greek	37 (5.4)	83 (13.5)	
Smoking status, n (%)			0.691
Smoker	236 (35.3)	214 (36.2)	
Non-smoker	432 (64.7)	377 (63.8)	
Parity, n (%)			0.265
Primiparous	304 (43.6)	240 (39.9)	
Multiparous	394 (56.4)	362 (60.1)	
Pre-pregnancy BMI (kg/m^2), mean (SD)	24.4±4.7	23.8±4.8	0.002
Pre-pregnancy BMI categories			0.639
< 25 kg/m^2	459 (68.0)	417 (69.4)	
≥ 25 kg/m^2	216 (32.0)	184 (30.6)	
Gestational weight gain (kg), n (%)	13.8±5.6	14.1±6.4	0.743
Delivery type, n (%)			0.454
Vaginal	339 (49.4)	313 (51.6)	
Caesarean	347 (50.6)	293 (48.3)	
**Child characteristics**			
Child gender, n (%)			0.226
Male	358 (52.0)	302 (48.8)	
Female	331 (48.0)	317 (51.2)	
Breastfeeding (months), n (%)			0.014
Ever	578 (87.2)	436 (82.1)	
Never	85 (12.8)	95 (17.9)	
Birth weight (kg), mean (SD)	3.20±0.4	3.14±0.4	0.030
Gestational age (weeks), mean (SD)	38.2±1.5	38.2±1.6	0.923
**Contaminants**	**Participants n=689 Median (IQR)**	**Non-Participants n=433 Median (IQR)**	
HCB	82.53 (54.86)	81.37 (61.16)	0.842
DDE	1981.19 (2326.54)	2096.96 (2636.32)	0.487
Total PCBs	319.42 (266.39)	258.5 (233.49)	<0.001

[a]Statistically significant differences ($p<0.05$), based on Mann-Whitney U test for two independent samples and Pearson's $\chi 2$ test for independence.

Table S2. Maternal and child characteristics by concentrations of first trimester maternal serum POPs levels (pg/ml), Mother-child cohort "Rhea" in Crete, Greece (n=689).

Characteristic	N	HCB r^a or Mean ± SD	DDE r^a or Mean ± SD	PCBs r^a or Mean ± SD
Maternal characteristics				
Maternal age (years)	681	0.53*	0.43*	0.67*
<20	18	52.7±21.0	1097.8±732.2	120.8±80.9
≥20-30	276	79.2±68.2	2068.3±2167.5	263.4±176.3
≥30-40	368	125.5±95.0	3533.3±3312.1	470.7±292.4
≥40	19	289.1±285.9	6223.8±3656.4	688.9±409.0
Pre-pregnancy BMI (kg/m^2)	675	0.18*	0.10*	-0.07
Underweight (<18.5)	22	61.4±33.9	1864.1±1515.9	306.2±234.2
Normal (≥18.5-25)	437	105.0±104.6	2825.8±2733.2	400.5±295.7
Overweight (≥25-30)	137	118.8±95.0	3347.1±3525.8	371.1±240.6
Obese (≥30)	79	133.3±110.0	2927.5±2980.4	317.3±170.6
Weight gain during pregnancy (kg)	566	-0.10*	-0.12*	-0.04
Ethnic origin				
Other	37	113.5±110.6	2948.3±3235.7	362.9±269.6
Greek	648	109.0±101.4	2936.4±2980.8	385.1±283.0
Education				
Low	105	98.4±75.7	2663.3± 2827.4	298.0± 233.9
Medium	347	100.8±86.6	2737.0 ± 2842.7	379.0± 316.3
High	226	128.1±129.6	3295.7±3047.0	423.9±197.2
Parity				
Primiparous	284	94.8±64.5	2496.3±2364.8	362.6±324.9
Multiparous	378	116.4±104.6	3255.6±3390.5	397.1±241.6
Smoking during pregnancy				
Never	432	105.2±90.3	2860.4±2773.9	372.7±239.6
Ever	236	117.0±122.2	2930.2±3096.5	395.5±325.1

Characteristic	N	HCB<br>r^a or Mean ± SD	DDE<br>r^a or Mean ± SD	PCBs<br>r^a or Mean ± SD
Type of delivery				
Vaginal	339	103.2±83.0	2752.7±2760.1	364.2±214.6
Caesarian	347	115.2±117.2	3174.0±3370.4	408.5±344.9
Breastfeeding (months)	663	0.001	0.04	0.09*
Never	85	140.9±148.5	3109.5±3062.1	389.2±236.1
Ever	578	105.6±94.1	2966.9±3142.9	384.4±281.8
BMI at child's 4 years	662	0.16*	0.11*	-0.09*
Underweight (<18.5)	20	87.0±52.7	2024.2±1705.2	371.3±222.3
Normal (≥18.5-25)	337	100.6±91.7	2679.3±2587.7	405.0±267.4
Overweight (≥25-30)	201	116.9±115.1	3300.0±3196.8	373.1±314.4
Obese (≥30)	104	132.4±117.9	3016.3±3336.3	332.6±211.7
Child characteristics at birth				
Sex				
Boy	358	107.3±98.8	3023.9±3134.7	375.2±294.0
Girl	331	111.1±104.7	2888.3±3032.1	397.5±281.8
Birth weight (g)	686	-0.09*	-0.07*	-0.04
Gestational age (completed weeks)	686	-0.08*	-0.12*	-0.03
Weight at 4 years (kg)	689	0.12*	0.07	0.02
Child characteristics at 4 years				
Height (cm)	689	0.11*	0.04	0.03
BMI (kg/m^2)	689	0.08*	0.05	-0.01
Triceps skinfold thickness (mm)	657	0.07	0.04	0.06
Subscapular skinfold thickness (mm)	661	0.07	0.06	0.01
Suprailiac skinfold thickness (mm)	643	0.05	0.05	0.04
Quadriceps skinfold thickness (mm)	621	0.10	0.06	0.04
Sum of skinfolds (mm)	668	0.09*	0.03	0.06
Waist circumference (cm)	683	0.14*	0.12*	0.06
Systolic blood pressure (mmHg)	552	0.11*	0.09*	0.04

**Characteristic**	**N**	**HCB**<br>**r[a] or Mean ± SD**	**DDE**<br>**r[a] or Mean ± SD**	**PCBs**<br>**r[a] or Mean ± SD**
Diastolic blood pressure (mmHg)	552	0.08	0.09*	0.02
Total Cholesterols (mg/dl)	588	0.02	0.06	0.06
HDL Cholesterol (mg/dl)	588	-0.03	0.02	-0.07
LDL Cholesterols (mg/dl)	588	0.02	0.05	0.07
C-reactive protein (ml/dl)	540	0.06	0.09*	0.03
Leptin (ng/ml)	580	0.11*	0.12*	0.07
Adiponectin (ul/ml)	581	-0.04	0.03	-0.05

[a]Spearman rho.

*p<0.05 for Spearman correlation.

Table S3. Associations of in utero POPs exposure with child lipids and adiponectin levels at 4 years of age, Mother-child cohort "Rhea" in Crete, Greece.

Outcome	N	Exposure[a]	Adjusted Model[b]
Total Cholesterol (mg/dl) [β (95% CI)]	448	HCB	-0.54 (-13.26, 12.18)
		DDE	4.44 (-3.35, 12.23)
		PCBs	6.85 (-7.48, 21.17)
HDL Cholesterol (mg/dl) [β (95% CI)]	448	HCB	-0.14 (-5.12, 4.84)
		DDE	2.77 (-0.28, 5.81)
		PCBs	-2.44 (-8.05, 3.17)
Adiponectin (ul/ml) [β (95% CI)]	442	HCB	0.82 (-3.14, 4.77)
		DDE	1.82 (-0.61, 4.24)
		PCBs	-2.10 (-6.57, 2.37)

[a]POP concentrations are $\log_{10}$ transformed, in pg/ml. [b]Adjusted for maternal serum triglycerides and cholesterol, maternal age, pre-pregnancy BMI, parity, maternal educational level, smoking status during pregnancy, breastfeeding duration, child sex, birth weight, gestational age and exact age at 4 year examination.

Table S4. Association between POPs and birth outcomes in multipollutant models including DDE, HCB and PCBs.

**Outcome**	**Exposure**[a]	**Adjusted Model**[b]
Rapid Growth 0-6 months[c] [RR (95% CI)]	HCB	1.88 (0.90, 3.99)
	DDE	1.20 (0.79, 1.88)
	PCBs	0.83 (0.37, 1.83)
BMI z-score at 4 years [β (95% CI)]	HCB	0.41 (-0.004, 0.82)
	DDE	0.20 (-0.05, 0.45)
	PCBs	-0.07 (-0.53, 0.39)
Obesity at 4 years [RR (95% CI)]	HCB	5.78 (1.13, 29.50)
	DDE	2.87 (0.86, 9.52)
	PCBs	1.16 (0.13, 10.47)
WC >=90th percentile[d] [RR (95% CI)]	HCB	2.10 (0.74, 5.95)
	DDE	2.71 (1.39, 5.28)
	PCBs	0.86 (0.23, 3.21)
Sum of skinfolds (mm) at 4 years [β (95% CI)]	HCB	6.35 (-0.01, 12.70)
	DDE	1.04 (-2.84, 4.91)
	PCBs	2.41 (-6.78, 9.50)
Systolic blood pressure (mmHg) [β (95% CI)]	HCB	3.66 (-0.52, 7.83)
	DDE	1.60 (-0.97, 4.18)
	PCBs	-0.39 (-5.05, 4.27)
Diastolic blood pressure (mmHg) [β (95% CI)]	HCB	2.53 (-0.40, 5.45)
	DDE	1.72 (-0.08, 3.53)
	PCBs	-2.59 (-5.86, 0.68)
C-reactive protein >3 mg/L [RR (95% CI)]	HCB	1.78 (0.43, 7.42)
	DDE	1.76 (0.70, 4.43)
	PCBs	2.57 (0.43, 15.40)
Leptin (ng/ml) [β (95% CI)]	HCB	1.59 (-0.32, 3.50)
	DDE	0.84 (-0.29, 1.98)
	PCBs	0.43 (-1.72, 2.58)

[a]POP concentrations are $\log_{10}$ transformed, in pg/ml. [b]Adjusted for maternal serum triglycerides and cholesterol, maternal age, pre-pregnancy BMI, parity, maternal educational level, smoking status during pregnancy, breastfeeding duration, child sex, birth weight, gestational age and exact age at 4 year examination. [c]Rapid growth models are adjusted for maternal serum triglycerides and cholesterol, maternal age, pre-pregnancy BMI, parity, maternal educational level, smoking status during pregnancy, breastfeeding duration, child sex and gestational age. [d]WC≥58.6cm.

Table S5. Associations of in utero POPs exposure with child BMI at 4 years of age in the subgroups defined by infant sex, maternal smoking and maternal prepregnancy BMI status.

POP and infant subgroup	N	z-BMI[a] β (95% CI)
HCB (log_{10} pg/ml)		
All	531	0.49 (0.12, 0.86)
Boys	267	0.37 (-0.18, 0.92)
Girls	264	0.58 (0.06, 1.11)
p-interaction		0.141
Non-smokers	355	-0.43 (-0.05, 0.90)
Smokers	176	0.31 (-0.31, 0.93)
p-interaction		0.192
Pre-pregnancy BMI <25 kg/m^2	357	0.62 (0.20, 1.04)
Pre-pregnancy BMI ≥25 kg/m^2	174	0.01 (-0.77, 0.79)
p-interaction		0.973
Inadequate GWG*	100	0.86 (0.04, 1.70)
Adequate GWG*	156	0.40 (-0.31, 1.10)
Excessive GWG*	193	0.17 (-0.46, 0.80)
p-interaction		0.470/0.128
DDE (log_{10} pg/ml)		
All	531	0.27 (0.04, 0.51)
Boys	267	0.33 (-0.01, 0.67)
Girls	264	0.25 (-0.08, 0.57)
p-interaction		0.730
Non-smokers	355	0.27 (-0.01, 0.56)
Smokers	176	0.17 (-0.26, 0.59)
p-interaction		0.467
Pre-pregnancy BMI <25 kg/m^2	357	0.33 (0.06, 0.60)
Pre-pregnancy BMI ≥25 kg/m^2	174	-0.05 (-0.50, 0.40)
p-interaction		0.894
Inadequate GWG*	100	0.45 (-0.10, 1.00)
Adequate GWG*	156	0.32 (-0.12, 0.75)
Excessive GWG*	193	0.31 (-0.10, 0.72)
p-interaction		0.905/0.480
PCBs (log_{10} pg/ml)		
All	531	0.21 (-0.20, 0.63)
Boys	267	0.30 (-0.27, 0.87)
Girls	264	0.21 (-0.41, 0.83)
p-interaction		0.550
Non-smokers	355	0.19 (-0.31, 0.69)
Smokers	176	0.17 (-0.62, 0.95)
p-interaction		0.276
Pre-pregnancy BMI <25 kg/m^2	357	0.03 (-0.46, 0.51)
Pre-pregnancy BMI ≥25 kg/m^2	174	0.35 (-0.46, 1.17)

POP and infant subgroup	N	z-BMI[a]<br>β (95% CI)
p-interaction		0.172
Inadequate GWG*	100	0.87 (-0.11, 1.85)
Adequate GWG*	156	-0.15 (-0.95, 0.64)
Excessive GWG*	193	0.13 (-0.63, 0.88)
p-interaction		0.674/0.264

[a]Adjusted for maternal serum triglycerides and cholesterol, maternal age, pre-pregnancy BMI, parity, maternal educational level, smoking status during pregnancy, breastfeeding duration, child sex, birth weight, gestational age and exact age at 4 year examination.

*Gestational weight gain (GWG) categories: inadequate, adequate and excessive, according to The Institute of Medicine (IOM) guidelines of 2009 based on pre-pregnancy BMI; Rasmussen K.M., Yaktine A.L. (Eds.), Weight gain during pregnancy: reexamining the guidelines, National Academies Press, Washington, DC (2009).

Table S6. Associations of in utero POP exposures with the outcomes under study in the total population and without out preterm births and low birth weight neonates[a].

Outcome	Exposure	Total population	Excluding preterm newborns	Excluding low birth weight newborns
Rapid Growth 0-6 months [RR (95% CI)]	HCB	1.94 (0.99, 3.77)	2.21 (1.03, 4.77)	1.79 (-0.89, 3.62)
	DDE	1.33 (0.89, 1.99)	1.31 (0.81, 2.11)	1.38 (0.90, 2.12)
	PCBs	1.20 (0.59, 2.46)	1.19 (0.51, 2.79)	1.15 (0.54, 2.47)
BMI z-score at 4 years [β (95% CI)]	HCB	0.49 (0.12, 0.86)	0.56 (0.18, 0.95)	0.54 (0.17, 0.92)
	DDE	0.27 (0.04, 0.51)	0.33 (0.08, 0.57)	0.30 (0.06, 0.54)
	PCBs	0.21 (-0.20, 0.63)	0.21 (-0.23, 0.65)	0.24 (-0.18, 0.67)
Obesity at 4 years [RR (95% CI)]	HCB	8.14 (1.85, 35.81)	11.8 (2.62, 53.33)	8.36 (1.89, 37.06)
	DDE	3.80 (1.19, 12.14)	3.83 (0.96, 15.27)	3.69 (1.15, 11.88)
	PCBs	3.91 (0.49, 31.27)	6.76 (0.64, 71.97)	3.70 (0.45, 30.18)
WC >=90th percentile [RR (95% CI)]	HCB	3.49 (1.08, 11.28)	3.21 (1.15, 8.94)	2.61 (1.01, 6.72)
	DDE	3.76 (1.70, 8.30)	2.67 (1.35, 5.28)	2.61 (1.38, 4.94)
	PCBs	2.08 (0.65, 6.71)	1.11 (0.30, 4.15)	1.64 (0.50, 5.37)
Sum of skinfolds (mm) at 4 years [β (95% CI)]	HCB	7.71 (2.04, 13.39)	8.43 (2.67, 14.19)	8.05 (2.18, 13.92)
	DDE	2.75 (-0.86, 6.35)	3.64 (-0.11, 7.39)	3.31 (-0.42, 7.03)
	PCBs	5.74 (-0.68, 12.16)	4.99 (-1.65, 11.63)	6.34 (-0.28, 12.97)
Systolic blood pressure (mmHg) [β (95% CI)]	HCB	4.34 (0.63, 8.05)	4.16 (0.34, 7.98)	4.68 (0.93, 8.43)
	DDE	2.31 (-0.07, 4.69)	2.99 (0.44, 5.53)	2.51 (0.09, 4.93)
	PCBs	2.16 (-2.03, 6.34)	1.41 (-3.01, 5.83)	2.41 (-1.83, 6.64)
Diastolic blood pressure (mmHg) [β (95% CI)]	HCB	2.48 (-0.13, 5.09)	2.40 (-0.33, 5.13)	2.49 (-0.16, 5.13)
	DDE	1.79 (0.13, 3.46)	1.72 (-0.10, 3.54)	1.92 (0.22, 3.61)
	PCBs	-0.49 (-3.43, 2.45)	-1.01 (-4.16, 2.14)	-0.17 (-3.15, 2.81)
C-reactive protein >3 mg/L [RR (95% CI)]	HCB	2.88 (0.86, 9.64)	2.77 (0.78, 9.80)	2.97 (0.88, 10.01)
	DDE	2.23 (0.94, 5.29)	2.56 (1.04, 6.27)	2.42 (1.02, 5.78)
	PCBs	4.50 (0.89, 22.76)	3.21 (0.59, 17.47)	4.15 (0.81, 21.23)
Leptin (ng/ml) [β (95% CI)]	HCB	2.15 (0.42, 3.89)	2.39 (0.55, 4.24)	2.40 (0.60, 4.19)
	DDE	1.21 (0.16, 2.27)	1.20 (0.05, 2.35)	1.19 (0.09, 2.29)
	PCBs	1.55 (-0.42, 3.52)	1.43 (-0.72, 3.58)	1.42 (-0.61, 3.46)

[a]n of excluded children born preterm ranges from 66 to 45 and children born with low birth weight from 23 to 18 depending on the outcomes.
